# *Trichophyton rubrum* Elicits Phagocytic and Pro-inflammatory Responses in Human Monocytes Through Toll-Like Receptor 2

**DOI:** 10.3389/fmicb.2019.02589

**Published:** 2019-11-21

**Authors:** Giovanna Azevedo Celestrino, Ana Paula Carvalho Reis, Paulo Ricardo Criado, Gil Benard, Maria Gloria Teixeira Sousa

**Affiliations:** ^1^Laboratory of Medical Mycology-LIM-53, Clinical Dermatology Division, Hospital das Clínicas FMUSP, Instituto de Medicina Tropical de São Paulo, Universidade de São Paulo, São Paulo, Brazil; ^2^Centro Universitário Saúde ABC, Santo André, Brazil

**Keywords:** dermatophytosis, toll-like receptor 2, innate immune, *Trichophyton rubrum*, monocytes, fungal cell wall

## Abstract

Dermatophytosis is a superficial fungal infection mostly restricted to keratinized tissues such as skin, hair, and nails but with potential to cause invasive or even systemic disease in immunocompromised patients. *Trichophyton rubrum* is the main etiologic agent, accounting for approximately 80% of the cases. Mononuclear phagocytes respond to pathogens through phagocytosis followed by production of several antimicrobial molecules, such as reactive oxygen and nitrogen species, and failure in doing so may contribute to development of chronic fungal infections. Toll-like receptors (TLRs) located on the surface of phagocytic cells bind either directly to target particles or through opsonizing ligands and trigger an actin-mediated ingestion. Even though the mechanisms involved in TLR-mediated cytokine responses are well established, the contribution of TLR in the recognition of *T. rubrum* by adherent monocytes remains unclear. Here, we report that phagocytosis of *T. rubrum* conidia by adherent monocytes is mediated by TLR2. Blockade of TLR2 by neutralizing antibodies impaired the fungicidal activity of monocytes as well their secretion of tumor necrosis factor (TNF)-α, but neither nitric oxide (NO) production nor interleukin (IL)-10 secretion was disturbed. So far, our data suggest that TLR2 is required for efficient conidial phagocytosis, and the absence of TLR2 signaling in human monocytes may impair the subsequent inflammatory response. These findings expand our understanding of phagocyte modulation by this important fungal pathogen and may represent a potential target for interventions aiming at enhancing antifungal immune responses.

## Introduction

Dermatophytosis is a superficial fungal infection that is usually limited to the keratinized layer of the epidermis, hair, and nails, and whose main etiologic agent is *Trichophyton rubrum*, accounting for approximately 80% of the cases (Svejgaard and Nilsson, 2004; Romano et al., 2005; Godoy-Martinez et al., 2009; Lee et al., 2015). In immunocompromised patients, however, dermatophytes proliferate beyond the superficial layers, potentially leading to severe systemic infection with deep organ involvement (Jones et al., 1973; Teklebirhan and Bitew, 2015).

Cells of the innate immunity, such as dendritic cells, macrophages, and monocytes, recognize fungi by identifying components of the fungal cell wall using pattern recognition receptors (PRRs) on their surface. Toll-like receptors (TLRs), C-type lectins (CLRs), complement, and immunoglobulin Fc receptors are important PRRs in anti-fungal immunity (Hardison and Brown, 2012; Dambuza and Brown, 2015). Yoshikawa et al. (2016), for example, using a murine model of deep dermatophytosis, demonstrated that the absence of the CLRs Dectin-1 and Dectin-2 promoted an inefficient proinflammatory response against *T. rubrum* infection characterized by lower production of TNF-α and IL-1β by spleen cells, impairing disease resolution (Yoshikawa et al., 2016).

TLR2 is expressed in monocytes, neutrophils, and macrophages and recognizes various ligands in the fungal cell wall, e.g., mannan and phospholipomannan. In some fungal infections such as aspergillosis, TLR2 activation promotes increased phagocytosis and cytokine production (Chai et al., 2009). However, because TLR2 can form heterodimers with other receptors, triggering different signaling pathways (Oliveira-Nascimento et al., 2012), its contribution to a specific infection cannot be accurately predicted or can be only marginal, as observed for *Cryptococcus neoformans* infection (Nakamura et al., 2006).

*In situ* models showed that TLR2 expression was preserved in the epidermis of patients with localized or disseminated dermatophytosis, while TLR4 was poorly expressed in those patients (de Oliveira et al., 2015). Other *in vitro* systems, however, demonstrated that whole *T. rubrum* conidia could diminish TLR2 expression in a keratinocyte cell line (Huang et al., 2015). Thus, it is still unknown if and how TLR2 participates in the immunity to *T. rubrum*, particularly in the human context. Here, we investigated the role of TLR2 in the interaction of human monocytes with *T. rubrum* by examining the capacity of TLR2 to recognize *T. rubrum* conidia and to develop an inflammatory response following the fungal challenge.

## Materials and Methods

### *T. rubrum* Conidia Production

A clinical isolate of *T. rubrum* (IMT-20) was used in this study. Twelve-day-old cultures grown in potato dextrose agar medium at 25°C were prepared for conidia production. Conidia were collected, suspended in 0.9% NaCl solution, and filtered in 40 μm cell strainers (BD Biosciences) to remove hyphae fragments.

### Ethical Statement

Blood samples were collected from 11 healthy adult volunteers (5 males, 6 females, mean age 35 years) who were free of infectious or inflammatory diseases at the moment of sample acquisition. All donors provided written informed consent to participate in this study. This work was approved by the ethical committee of Clinics Hospital, University of São Paulo (approval number 065235/2018).

### Monocyte Isolation and Cultures

A total of 70 ml of peripheral blood was collected from each donor in heparin tubes. Peripheral blood mononuclear cells (PBMCs) were obtained by density gradient centrifugation using the commercial reagent Ficoll-Paque™ PLUS (GE Healthcare) as previously described by de Sousa et al. (2015). Monocytes were purified from PBMCs by adherence. Cells were plated on glass coverslips and incubated for 90 min to allow monocyte adherence, followed by washing with phosphate buffer saline (PBS) for removal of non-adherent cells.

Cells were cultured in culture medium [RPMI-1640 with Glutamax™ supplemented with pyruvate (0.02 mM), 100 U/ml penicillin, 100 mg/ml streptomycin (all from Sigma-Aldrich), and 10% human pooled serum] at 37°C, 95% humidity, and 5% CO_2_ in 24-well round-bottom plates. Monocytes were preincubated for 50 min with 10 μg/ml of TLR2-blocking antibody (anti-hTLR2-IgA, catalog code: maba2-htlr2) or its isotype control IgA1 (catalog code: maba2-ctrl, both from InvivoGen) before stimulation with *T. rubrum* conidia. The antibody’s blocking concentration was based on previous studies (van de Veerdonk et al., 2009).

### Phagocytosis Assay

After TLR2 blockade, monocytes (2 × 10^5^ cells) were incubated with *T. rubrum* conidia (10 × 10^5^) at 37°C and 5% CO_2_ in 24-well round-bottom plates (with coverslips) for 3 h. Coverslips were removed, washed with PBS at 37°C to remove non-adherent cells, fixed with methanol, stained with 20% Giemsa solution, and analyzed under an optical microscope. For transmission electron microscopy, the protocol from Campos et al. (2006) was used. The phagocytosis index (PI) was calculated by multiplying the percentage of monocytes in the field that phagocytosed at least one conidium by the mean number of phagocytosed particles. For colony-forming unit (CFU) assay, the above phagocytosis assay was performed without coverlips. After 6 h, wells were washed (three times) with PBS 1× to remove free fungi, and monocytes were lysed with 0.1% Triton X-100 solution. A 10-fold dilution of the samples was plated on Sabouraud Agar (Difco) and kept at 30°C up to 7 days. Recovered colonies were counted and multiplied by dilution factor.

### NO Measurements

The determination of NO production by monocytes was estimated by the measuring nitrite (${\mathrm{NO}}_{2}^{-}$) levels in the culture supernatants. Monocytes were incubated with *T. rubrum* conidia at 37°C and 5% CO_2_ for 3 h, and supernatants were collected. The accumulation of ${\mathrm{NO}}_{2}^{-}$ was quantified by the Griess method. Briefly, 50 μl of supernatant was incubated with an equal volume of the Griess reagent for 10 min at room temperature, and the absorbance was measured using a plate reader with a filter between 520 and 550 nm. ${\mathrm{NO}}_{2}^{-}$ concentration was determined by running in parallel a standard curve with concentrations ranging from 0 to 100 μM.

### Cytokine Measurements

The production of the cytokines TNF-α, IL-1β, IL-6, and IL-10 in the supernatants of monocytes cultured for 18 h was evaluated and quantified by ELISA, following the manufacturer’s instructions (BD Biosciences). *Escherichia coli* lipopolysaccharides (LPS; 100 ng/ml) and Zymosan (8 μg/ml) were used as the positive control for cytokine release, both from Sigma-Aldrich. The results were expressed in pg/ml and determined from standard curves established for each assay.

### Statistical Analysis

Data were expressed as mean ± SEM and analyzed in the software GraphPad Prism (version 6.00 for Windows, GraphPad Software, San Diego, CA, USA; www.graphpad.com) by two-way ANOVA and Bonferroni *post hoc* test. All experiments were performed at least three times, and data were presented as the mean ± SEM of all experiments performed, unless otherwise indicated.

## Results

### Phagocytosis of *T. rubrum* Is Less Efficient in the Absence of Toll-Like Receptor 2 Activation

**Figure 1:**
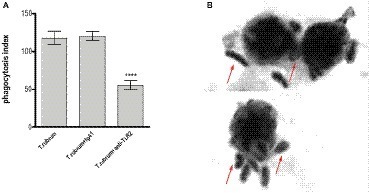
TLR2 blockade leads to impaired phagocytosis by human monocytes. Monocytes treated with TLR2 neutralizing antibody (or isotype control) were co-cultured with *T. rubrum* conidia at a ratio of 1:5 for 3 h, fixed and stained with Giemsa solution. Conidia internalization was counted by optical microscopy, and the phagocytosis index (PI) was calculated. **(A)** PI of *T. rubrum* conidia by monocytes. **(B)** Images of *T. rubrum* conidia phagocytosis by monocytes (without TLR2 blockade) were analyzed by optical microscopy (1,000×), and arrows indicate fungal structures. Two-way ANOVA and Bonferroni *post hoc* test: ^****^p < 0.0001. Microscopy photos taken from one representative of 11 independent experiments, each performed in triplicate, are shown.

**Figure 2:**
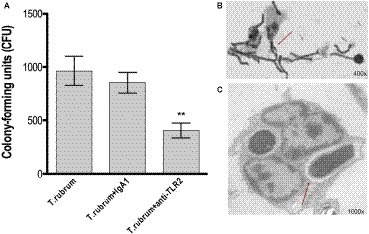
TLR2 blockade decreases fungicidal activity by monocytes. Monocytes treated with TLR2 neutralizing antibody (or isotype control) were incubated with *T. rubrum* conidia at a ratio of 1:5 for 3 h. **(A)** Fungal loads in monocytes were determined by CFU assay. Images of the interaction between monocytes (without TLR2 blockade) and *T. rubrum* conidia analyzed by optical (400×) **(B)** and transmission electron microscopy (1,000×) **(C)** after 24 hours. Arrows indicate fungal structures. Data are expressed as mean ± SEM. Two-way ANOVA and Bonferroni *post hoc* test: ^**^p < 0.01. Microscopy photos taken from one representative of four independent experiments, each performed in triplicate, are shown.

TLR2 recognizes various microbial ligands (e.g., phospholipomannan, zymosan, and glucuronoxylomannan) and makes use of different mechanisms to provide specificity to each of them (Oliveira-Nascimento et al., 2012). We first investigated whether TLR2 can be involved in the phagocytosis of *T. rubrum* conidia by human monocytes by determining the PI after 3 h of interaction between phagocytes and fungal particles. Thus, our results showed that while monocytes efficiently ingested *T. rubrum* conidia (117.6 ± 8.6), functional blockade of TLR2 significantly inhibited fungal engulfment (55.5 ± 5.9; Figure 1). Consistent with this, the fungicidal activity of monocytes (852.8 ± 97.6) was also impaired when this receptor was blocked (405.5 ± 68.9; Figure 2A).

Taken together, these results indicate that TLR2 may be involved in the uptake and killing of *T. rubrum* by monocytes.

### Outcome of *T. rubrum* Infection in Monocytes

To determine the fate of *T. rubrum* after its uptake by monocytes, we analyzed the interaction of monocytes and *T. rubrum* conidia in different times (3, 6, 12, and 24 h) by using light and transmission electron microscopy. The results showed that *T. rubrum* conidia developed into hyphae after 24 h of incubation with monocytes (Figures 2B,C), reinforcing the notion that monocytes present poor fungicidal activity.

### Toll-Like Receptor 2 Does Not Participate in NO Production but Promotes Production of Pro-inflammatory Cytokines After *T. rubrum* Infection

**Figure 3:**
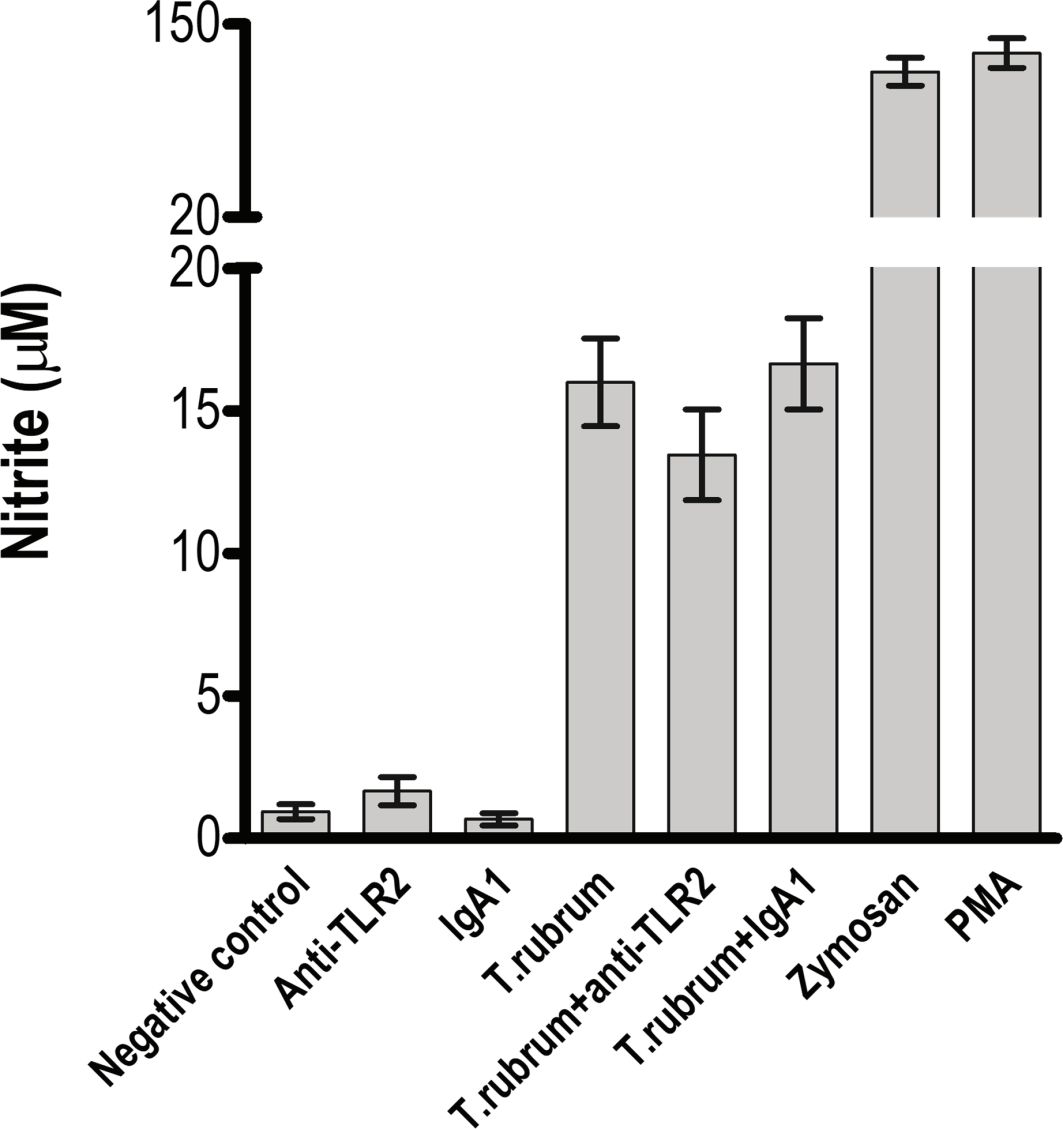
NO release by monocytes is not impaired by TLR2 blockade. Monocytes treated with TLR2 neutralizing antibody (or isotype control) were incubated with *T. rubrum* conidia at a ratio of 1:5. Supernatants were collected after 3 h, and ${\mathrm{NO}}_{2}^{-}$ was measured by the Griess method. The results are expressed as mean ± SEM (*n* = 11), each performed in triplicate. Two-way ANOVA and Bonferroni *post hoc* test.

**Figure 4:**
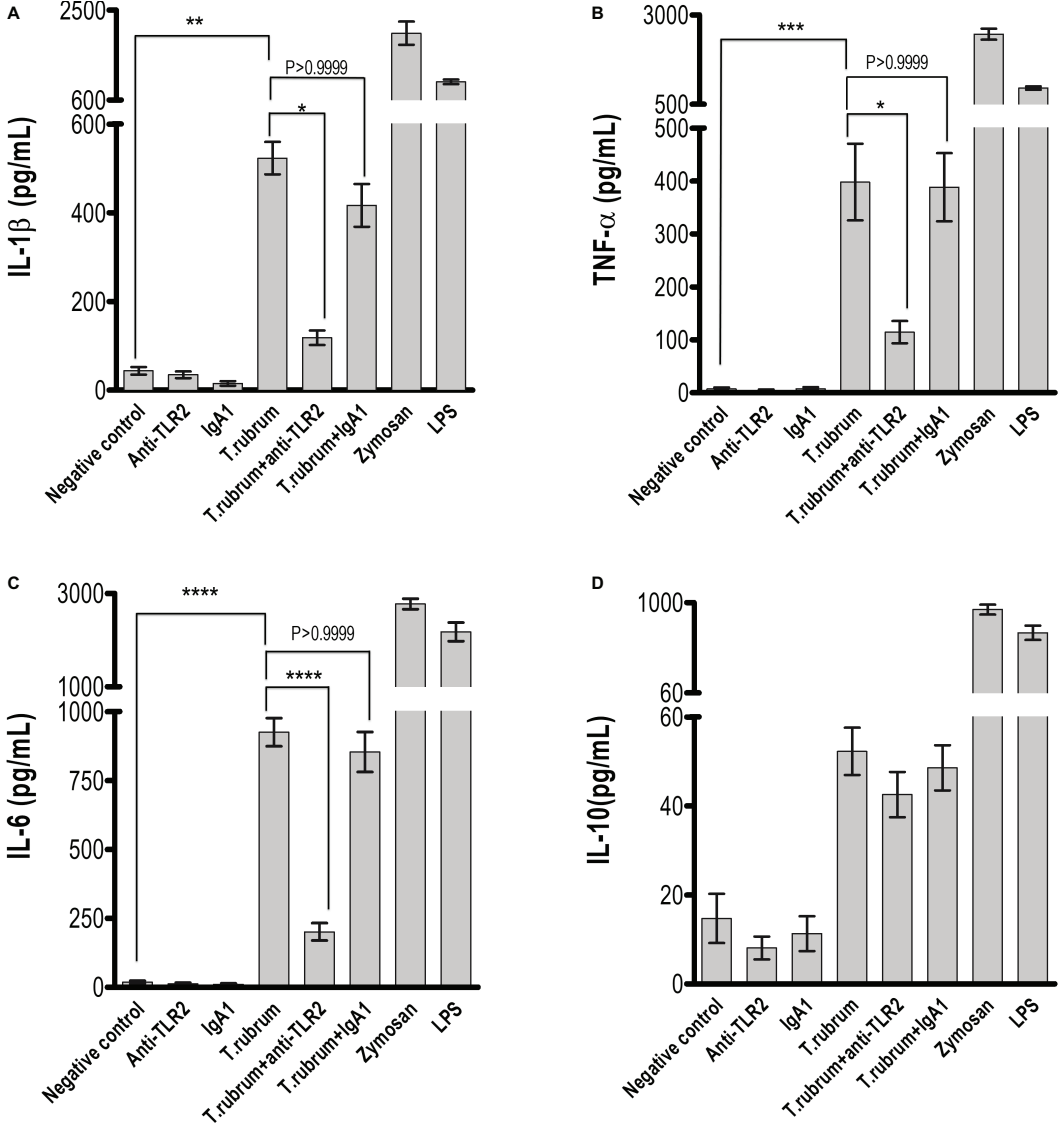
TLR2 blockade impaired pro-inflammatory but not regulatory IL-10 cytokine production by monocytes. **(A)** IL-1β, **(B)** TNF-α, **(C)** IL-6, and **(D)** IL-10 levels were quantified in the culture supernatants by ELISA after 18 h of *T. rubrum* addition. The results are expressed as mean ± SEM (*n* = 11), each performed in triplicate. Two-way ANOVA and Bonferroni *post hoc* test: ^*^p < 0.05; ^**^p < 0.01; ^***^p < 0.001; ^****^p < 0.0001.

**Figure 5:**
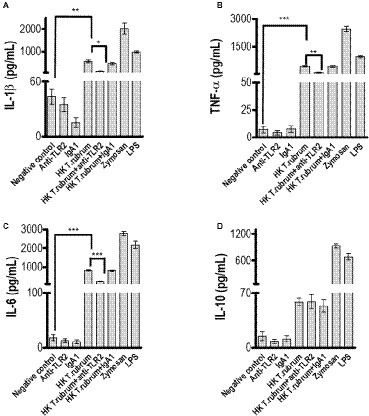
TLR2 blockade impaired pro-inflammatory but not regulatory IL-10 cytokine production by monocytes after infection with HK *T. rubrum* conidia. **(A)** IL-1β, **(B)** TNF-α, **(C)** IL-6, and **(D)** IL-10 levels were quantified in the culture supernatants by ELISA after 18 h of *T. rubrum* addition. The results are expressed as mean ± SEM (*n* = 11), each performed in triplicate. Two-way ANOVA and Bonferroni *post hoc* test: ^*^p < 0.05; ^**^p < 0.01; ^***^p < 0.001.

Next, we determined whether NO secretion by monocytes 3 h after fungal addition could also be TLR2 dependent. Albeit *T. rubrum* did not promote an oxidative burst as strong as zymosan or phorbol myristate acetate (PMA), we did observe NO production in our cultures (16 ± 1.5; Figure 3). However, TLR2 blocking did not alter the response (13.5 ± 1.6). Curiously, Campos et al. (2006) showed that murine macrophages did not secret NO (Campos et al., 2006), suggesting important differences between human and mice models of dermatophytosis. Finally, we analyzed the cytokine response upon TLR2 blockade. Thus, as shown in Figures 4A–C, infected monocytes produced significantly higher levels of TNF-α (398.0 ± 72.2 pg/mL), IL-6 (925.4 ± 51.0 pg/mL) and IL-1β (523,1 ± 36,5 pg/mL) as compared to TLR2 blocked counterparts (respectively, 114,5 ± 21.0 pg/mL, 201,3 ± 31,31 pg/mL, 118,4 ± 16,3 pg/mL). IL-10 secretion (52,2 ± 5,3 pg/mL), however, was not significantly affected by antibody addition (42,6 ± 5,1 pg/mL) (Figure 4D). Similar results were obtained in experiments with heat killed (HK) *T. rubrum* conidia as shown in Figures 5A–C, TNF-α (447,5 ± 48,7 pg/mL), IL-6 (829,2 ± 48,2 pg/mL) and IL-1β (563,8 ± 59.0 pg/mL) secretion were significantly higher compared to TLR2 blocked group, (respectively, 101.0 ± 21,1 pg/mL, 237,8 ± 26,3 pg/mL, 119,4 ± 13,7 pg/mL). However, IL10 (58,6 ± 4,9 pg/mL) release was not significantly altered with TLR2 blockade in presence of HK *T. rubrum* conidia (59.0 ± 9.0 pg/mL) (Figure 5D). Previous studies indicate that *T. rubrum*-infected murine macrophages secreted significant levels of IL-10 and TNF-α (Campos et al., 2006), but here we showed that these responses may require the concomitant signaling of other uncoupled receptors. Zymosan and LPS were used as positive controls. The amounts elicited in the positive control wells (LPS or Zymosan) were significantly higher than in the non-stimulated wells (medium only) for every cytokine.

## Discussion

Dermatophytosis is a chronic fungal disease, usually benign but difficult to treat. Few studies, however, have addressed the role of monocytes during the initial phase of this infection. Human and experimental studies have shown that TLRs have an essential function during infectious diseases, particularly fungal infections (Maisonneuve et al., 2014), while preclinical and clinical studies have explored the use of TLR agonists as adjuvants to reinforce the adaptative immune response during vaccination or treatment of infectious diseases (de Sousa et al., 2014; Ashour, 2015). In a previous report, we provided evidence that TLR2 participates in the host’s epidermis response to dermatophytosis (de Oliveira et al., 2015). These studies suggested that the preserved expression of TLR2 *in situ* acted to limit the inflammatory process and to preserve the epidermal integrity. Alternatively, TLR2 sustained expression could also contribute to the limited inflammation and persistent infection that are characteristic of some dermatophytic infections (de Oliveira et al., 2015).

Previously regarded as superficial fungal infections limited to the keratinized layers of the epidermis, dermatophytic infections in immunocompromised patients may evolve to deep-seated or disseminated infections, implying the involvement of other compartments of the immune system (Lanternier et al., 2013). We thus aimed to investigate the role of TLR2 during the interaction of *T. rubrum* conidia with human monocytes.

TLR2-dependent uptake involves the recognition of fungal carbohydrates, but several studies exploring its role in different models of fungal infections showed conflicting results, suggesting both protective and non-protective effects, which could be explained by differences in the virulence of pathogens, the antigens, and the genetic background of the animals used (Oliveira-Nascimento et al., 2012), and also in differences between animal vs. human studies. In our system, blockade of TLR2 leads to impaired phagocytosis and fungicidal activity. But since TLR2 is not a phagocytic receptor by itself, the inefficient phagocytosis could be explained by impaired secretion of opsonizing factors by the phagocytes or by a coadjutant role of TLR2 in the function of others *bona fide* internalizing receptors, for example, Dectin-1 (Gantner et al., 2003).

Moreover, whether TLR2 is blocked or not, the ingested conidia differentiated into hyphae, growing and killing the monocytes after 12 h of culture, recapitulating results found in murine macrophage models (Campos et al., 2006). These results indicate that fungal cells are able to inhibit monocyte functions or induce suppressive cytokines that could favor fungal evasion from host response.

Indeed, we showed that TLR2 contributes to *T. rubrum*-induced production of proinflammatory cytokines, but it does not participate in the secretion of the anti-inflammatory cytokine IL-10. The modulatory effects were observed in adherent monocytes and could be induced with both heat-killed and live conidia. Our data support the hypothesis that the absence of TLR2 signals may influence the function of monocytes and could increase the susceptibility to dermatophytosis. It is known that TLR2 influences macrophages and neutrophil anti-fungal activity, mainly through an effect on TNF-α production (Blasi et al., 2005; Gil et al., 2005; Gil and Gozalbo, 2006).

It is well known that IL-10 plays an inhibitory role in monocytes and neutrophil activity against fungal pathogens. Interestingly, normal human monocytes challenged with *T. rubrum* conidia released only small amounts of IL-10. This is in agreement with data from other fungi where conidia were also poor inducer of IL-10 compared, e.g., with the levels of pro-inflammatory cytokines secreted concomitantly, or compared with the IL-10 levels induced with hyphae (Netea et al., 2003). In fact, we speculate that induction of TLR-2-mediated production of IL-10 by *T. rubrum* conidia in normal human monocytes could be counterbalanced by signaling through other receptors, such as TLR4. Several studies showed in both mice and human monocytes that TLR4, which recognizes fungal mannan residues, signaled to increase pro-inflammatory cytokine production while decreasing IL-10 production (Netea et al., 2003; Chai et al., 2011; Kawasaki and Kawai, 2014). This regulation would not take place in monocytes from recurrent dermatophytosis patients, which secrete significantly more IL-10 than normal controls or tinea pedis patients (de Sousa et al., 2015). This may also explain why TLR2 blockade alone did not result in an increase in IL-10 levels.

We are now planning studies aiming to investigate the contribution of other phagocytic receptors (such as Dectin-1, mannose, or other TLRs such as TLR4) and their crosstalk with TLR2 signaling in the response to *T. rubrum*.

Intriguingly, while previous works suggest that TLR2 regulates the release of antimicrobial reactive oxygen and nitrogen species by innate immune cells (Tessarolli et al., 2010; Pinke et al., 2016), we could not recapitulate those findings in our experimental system, observing no TLR2 contribution in *T. rubrum*-induced NO secretion. However, since *T. rubrum* did not induce a high peak of oxidative species (compared to zymosan particles), we can hypothesize that this fungus does not promote a strong oxidative burst in monocytes and TLR2 can be important in regulating intense oxidative stress events.

All together, these data suggest that TLRs play different roles depending on cell type expression during infectious diseases, reinforcing the relevance of studies regarding TLR function in different cells in dermatophytosis.

## Conclusion

In conclusion, our results demonstrate that TLR2 is important for the ingestion of *T. rubrum* conidia and the production of pro-inflammatory cytokines by human monocytes, uncovering an important branch of the innate immune response in the first step of dermatophyte pathogenesis.

## Data Availability Statement

The datasets generated for this study are available on request to the corresponding author.

## Ethics Statement

The studies involving human participants were reviewed and approved by Comissão de Ética para Análise de Projetos – CAAPesq, Hospital das Clínicas, Faculdade de Medicina, Universidade de São Paulo. The participants provided their written informed consent to participate in this study.

## Author Contributions

GC carried out the experiment with support from AR. PC contributed to the interpretation of the results. GB and MS contributed equally to this work with analyzing the results and writing the manuscript. All authors discussed the results and contributed to the final manuscript.

### Conflict of Interest

The authors declare that the research was conducted in the absence of any commercial or financial relationships that could be construed as a potential conflict of interest.
